# Long-term healthcare use of COVID-19 cases in 2020: a two-year follow-up in Stockholm, Sweden

**DOI:** 10.1080/07853890.2025.2580077

**Published:** 2025-10-31

**Authors:** Nicholas Baltzer, Pontus Hedberg, Sara Nordqvist Kleppe, Joakim Dillner, Anders Sönnerborg, Jan Albert, Kristoffer Strålin, Pär Sparén, Pontus Nauclér

**Affiliations:** ^a^ Division of Infectious Diseases, Department of Medicine, Karolinska Institutet, Stockholm, Sweden; ^b^ Department of Medical Epidemiology and Biostatistics, Karolinska Institutet, Stockholm, Sweden; ^c^ Department of Medicine, Huddinge, Karolinska Institutet, Stockholm, Sweden; ^d^ Department of Laboratory Medicine, Karolinska Institutet, Stockholm, Sweden; ^e^ Division of Infectious Diseases, Karolinska University Hospital, Stockholm, Sweden; ^f^ Department of Microbiology, Tumor and Cell Biology, Karolinska Institute, Stockholm, Sweden; ^g^ Department of Clinical Microbiology, Karolinska University Hospital, Stockholm, Sweden

**Keywords:** COVID-19, SARS-CoV-2, serologic tests, delivery of healthcare, public health, registries

## Abstract

**Background:**

There is limited data on whether SARS-CoV-2 infections will result in increased long-term use of general healthcare, potentially impacting healthcare systems and management. Exploring this, we investigated the healthcare use of individuals with a SARS-CoV-2 infection in 2020 over a period of two years, using comprehensive medical records.

**Methods:**

We followed a cohort of 365,354 individuals in Stockholm, Sweden, who had been tested with SARS-CoV-2 serology in 2020, for healthcare use during 2021/22. SARS-CoV-2 seropositive and seronegative individuals were matched 1:1 on age, sex, 2019 healthcare use, and date of last serology, and compared on healthcare use during 2021/22 using registry linkages. Seropositive individuals were stratified on hospitalization for COVID-19 in 2020. Individuals were compared for total healthcare use, measured as incidence rate rations (IRR), and healthcare type usage-or-not per month, measured as a difference-in-differences regression.

**Results:**

There were 272,918 seronegative and 73,814 seropositive subjects. Incidence rate ratios (IRRs) for primary healthcare use were 1.0, 1.16, and 0.98, for all, only hospitalized, and only non-hospitalized, seropositive individuals respectively. For outpatient specialist care IRRs were 0.96, 1.31, and 0.93. For inpatient care IRRs were 0.98, 1.19, and 0.95. Healthcare type usage-or-not per month showed no substantial differences, ranging from 0.01 to -0.01 in deviation. Increased healthcare use during follow-up was restricted to the seropositive individuals hospitalized for COVID-19 in 2020.

**Conclusion:**

There was no increase in healthcare use in the overall population from SARS-CoV-2 infections during 2020, suggesting there is no apparent need to adapt healthcare systems at scale for the COVID-19 aftermath.

## Introduction

More than 770 million confirmed COVID-19 cases and nearly seven million deaths have been reported to the World Health Organization [1]. Yet, questions remain regarding the long-term health consequences for individuals with previous severe acute respiratory syndrome coronavirus 2 (SARS-CoV-2) infections and the implications for healthcare systems [2]. Post-Acute COVID-19 Syndrome (PACS), Post-COVID-19 Condition (PCC), or Post-Acute Sequelae of COVID-19 (PASC) have been widely reported as sequelae, with studies noting varying prevalences ranging from 12.7%-45.3% [3–5]. COVID-19 has been recognized as a multi-organ disease, raising concerns regarding the public health and long-term healthcare burden after the pandemic [6]. Moderate to severe COVID-19 has been investigated for long-term health consequences, with reports of negative effects and multi-organ damage five months or more after the acute infection phase [3,7]. Treatment centres for PACS have been established to deal with chronic conditions that have arisen, but the healthcare consequences of the general population are unclear [8]. Many studies have focused on a hospital setting or a self-referral scenario, with limited generalizability to the wider public [2]. Studies using polymerase chain reaction (PCR) tests for ongoing infections [9], while SARS-CoV-2 serology captures previous infections including infections in asymptomatic individuals [10]. The median duration of SARS-CoV-2 viral shedding is around 18 days [11]. Seroconversion rates for SARS-CoV-2 are around 90% [12], and combined positivity for IgG, IgM and IgA has been reported at >90% even 616 days after infection [13]. Overall, using serology reduces misclassification for individuals with a prior SARS-CoV-2 infection.

The response to the COVID-19 pandemic differed over the world. Swedish authorities adopted a mitigation strategy aimed at curtailing the spread of infection [14]. No lockdowns were enacted, and preventive measures such as social distancing, working from home, and restrictions on larger gatherings, were left to voluntary adherence. Unlike other Nordic countries, grade schools did not switch to at-home schooling methods [15]. Visits to nursing homes and eldercare facilities were banned in March 2020 [14]. Routine healthcare, such as breast cancer screening, was postponed or cancelled. While ICU and inpatient capacity was almost overwhelmed in April-May 2020, field hospitals and medical tents expanded the availability. While the less stringent directives adopted by Sweden resulted in an elevated mortality during the initial waves of the pandemic, primarily among the older demographics, long-term excess mortality was similar to other Nordic countries [16]. Initially, the available testing capacity was focused on the hospitalized, healthcare staff, and vulnerable demographics [17]. By June 2020, testing capacity had been scaled up to more than 50,000 tests per week, and by September 2020, the capacity exceeded 100,000 [18]. Free-of-charge PCR testing, through clinics, mobile test stations, self-sampling kits, and temporary testing stations, were available to the public from June 2020 until February 2022 in all of Sweden [19]. Additionally, serology testing was provided free-of-charge in Stockholm from June 2020 until the end of May 2021, using the same distribution methods. Vaccinations became available late December 2020, and by mid-May 2021 87.2% of the population had been vaccinated [20].

To investigate the long-term healthcare consequences of SARS-CoV-2 infections, we performed a retrospective population-based cohort study in Stockholm County, Sweden, where SARS-CoV-2 serology testing was available free-of-charge, comparing healthcare use over two years of follow-up in antibody-positive and antibody-negative adults tested during the pre-vaccination period in 2020.

## Materials and methods

### Study population

**Figure 1.**
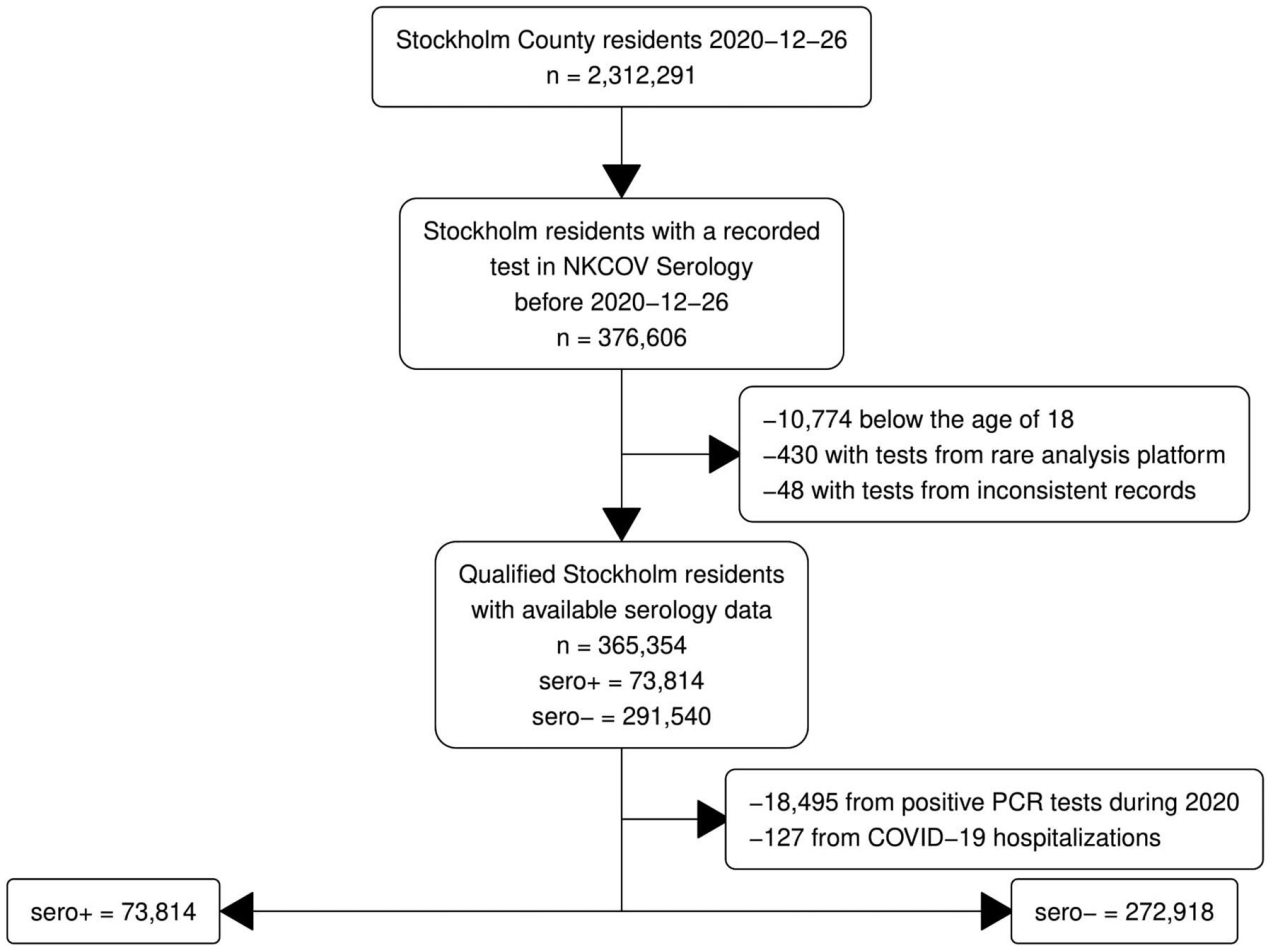
Flow chart for the study population. All residents of Stockholm in 2020 with a recorded serology test were considered. The individuals aged 18 or above with validated results were separated into seropositive (sero+) and seronegative (sero-) groups based on their test results, with seronegative individuals also being excluded from the study if any other source, such as PCR tests or hospital data, had designated them as SARS-CoV-2 positive at any point during 2020.

We identified all individuals in Stockholm County with a serology test taken in 2020, recorded in the SARS-CoV-2 national quality registry (NKCOV). NKCOV collects laboratory test results from all microbiological laboratories that performed analyses for SARS-CoV-2 in Stockholm County. All but one laboratory submitted data to the registry. Consent to participate in the study was waived by the Swedish Ethical Review Authority [21]. There were 2,312,291 individuals residing in Stockholm County between 2020 and 01–31 and 2020-12-26, the time from the first recorded COVID-19 case to initiation of the first COVID-19 vaccination program. Of these, 376,606 had at least one serology test recorded during 2020 (Figure 1). Positive and negative test results were determined by standard laboratory procedures in accredited laboratories for clinical microbiology (see appendix for test details). The study was limited to adults and 10,774 individuals <18 years old were excluded. To ensure that test results were verifiably accurate, an a priori decision was made to exclude tests performed on analysis platforms with fewer than 1,000 records in NKCOV (*n* = 430). Additionally, 48 records with incomplete data were excluded. From the remaining population of 365,354 individuals, 73,814 with a positive serology test recorded during 2020 were classified as the seropositive (sero+) group. From the 291,540 individuals with only negative serology tests during 2020, 18,495 had a positive PCR test recorded in either the Swedish registry of notifiable communicable diseases (SmiNet) or NKCOV and 127 individuals with a hospitalization or ICU visit with a COVID-19 discharge code during 2020 were excluded. The remaining 272,918 individuals were classified as the seronegative (sero-) group. In the sero + group, 67,450 individuals were included based on a test targeting the nucleocapsid and 6,364 were included on a test targeting the spike protein. In the sero- group, the corresponding numbers were 244,156 and 28,762.

### Study design

The study population was followed from 2020 to 12–27 until 2022-10-15. A matched cohort design was used to account for baseline differences between the sero + and sero- groups. Individuals were censored when moving out of the county or on death. Furthermore, we performed analyses with and without censoring individuals when they had a positive PCR SARS-CoV-2 test during follow-up to account for healthcare use induced by SARS-CoV-2 infections during 2021 and 2022.

### Data sources

Data were collected from the NKCOV registry, the Swedish Intensive Care Registry (SIR), the Stockholm regional healthcare data warehouse (VAL), SmiNet and Statistics Sweden. The data were linked using personal identification numbers and pseudonymised.

From SIR, an intensive care quality registry [22], we gathered data on all ICU admissions over the time period. From VAL, a database of administrative healthcare data in Stockholm county [23], we gathered demographics, migration, inpatient stays and outpatient visits reimbursed by Region Stockholm. VAL has near complete coverage of specialist care and includes 94% of all primary care data for the region. From Statistics Sweden we gathered sociodemographic data [24]. From SmiNet we gathered all PCR SARS-CoV-2-positive test results, data that must be registered in accordance with the Communicable Diseases Act [25].

### Outcome

The outcome of the study was healthcare use assessed as number of unique visits to doctors within primary care, outpatient specialist care, and inpatient care.

### Statistics

We first described baseline characteristics of the overall study population as well as the matched cohorts. Continuous variables were presented with median (interquartile range [IQR]) and categorical variables were reported as frequencies (percentage).

Healthcare use during the follow-up period was compared to usage during the pre-pandemic period of 2019. We assessed healthcare use during calendar years 2021, 2022, and in total over the full follow-up, as well as per month. Recorded healthcare visits were limited to one visit per day to account for administrative procedures and in-hospital movements between wards. Healthcare use was also computed per month (whether an individual had any recorded healthcare use in that month).

A matched cohort design was used to approximate similar starting points for healthcare use in 2019. Matching used a nearest neighbour algorithm on age, sex, Charlson Comorbidity Index Score (CCI) [26] and date of last serology test in 2020, with the additional requirement of an exact match on binned primary and inpatient healthcare use counts in 2019, using bins 0, 1–2, 3–5 and 6+. Covariate balance before and after matching was assessed using absolute standardized mean differences (SMDs). A difference-in-difference [27] (DiD) model was used to compare healthcare usage before and after 2020, and incidence rate ratios (IRR) were computed as quasi-Poisson mixed model regression [28] estimates with robust standard errors [29]. For the DiD model, The Equal Trends Assumption underlying the DiD model was assessed by visually inspecting monthly healthcare utilization trajectories by calendar month for sero + and sero– groups across primary, outpatient specialist, and inpatient care. Both IRR and DiD estimates were reported, as IRRs quantify relative healthcare utilization during follow-up, while DiD accounts for baseline utilization trends and isolates within-individual changes over time. Regression estimates were presented with 95% confidence intervals (95% CI).

Stratified analyses were performed according to if sero + individuals had been treated in hospital due to COVID-19 (defined as main discharge code COVID-19 for the hospitalization) in 2020. The stratified sero + individuals were compared to their respective sero- controls as previously described.

All statistical analyses were performed in R 4.1 [30]. Regressions were performed with the Stats package, and matching was done using MatchIt [31].

### Sensitivity analysis

To assess the sensitivity of the chosen inclusion criteria, we also performed analyses without excluding individuals from the sero- group based on positive PCR tests. This resulted in a sero- group consisting of 290,737 individuals. Further, we restricted an analysis to individuals with serology performed within the last month before follow-up (2020-11–26 until 2020-12-26) to reduce the risk of misclassification of exposure. This resulted in a matched cohort of 19,861 sero + individuals.

## Results

### Baseline characteristics

There were 73,814 individuals in the sero + group and 272,918 in the sero- group (Figure 1). The serology test distributions (using the last test for each individual) in 2020 were similar in the sero + and the sero- groups (Supplementary Figure 1). The first peak of COVID-19 hospitalizations occurred in April-May followed by a second peak in November-December of 2020. Most tests were performed during July-August when serology testing became available free-of-charge.

The age was higher in the sero- group: median age 47 (IQR 35–58), compared to 44 (IQR 31–55) in the sero + group (Table 1). Male sex was more common, 56.8% and 54.2%, for sero- and sero + respectively. Swedish origin and higher education level were more common in the sero- group. The comorbidity burden was higher in the sero- group, 12.2% vs 10.6% had a CCI of 1 or more. The sero- group had a higher consumption of primary care, outpatient specialist care, and inpatient care in 2019 (Table 1). In the matched cohorts, the distributions for the matching variables age, sex, primary healthcare use in 2019, inpatient healthcare use in 2019, and date of the last serology test in 2020 were similar for sero + and sero- individuals (Table 1). In total, 2,597 sero + individuals were hospitalized for COVID-19 in 2020.

**Table 1.** Baseline characteristics of the study population and matched cohorts by serology test result.

		Overall	sero-	sero+	SMD	Matched sero-	Matched sero+	SMD
N		346,732	272,918	73,814		73,814	73,814	
Person days (med IQR)		657[657, 657]	657[657, 657]	657[657, 657]	0.011	657[657, 657]	657[657, 657]	0.026
Age (med IQR)		46 [34, 57]	47 [35, 58]	44 [31, 55]	0.221	43 [31, 54]	44 [31, 55]	0.030
Age Category	18–39	126,692 (36.5)	95,791 (35.1)	30,901 (41.9)	0.223	32,020 (43.4)	30,901 (41.9)	0.050
	40–49	74,715 (21.5)	58,655 (21.5)	16,060 (21.8)		16,417 (22.2)	16,060 (21.8)	
	50–59	71,242 (20.5)	55,857 (20.5)	15,385 (20.8)		14,620 (19.8)	15,385 (20.8)	
	60–69	44,435 (12.8)	36,595 (13.4)	7,840 (10.6)		7,197 (9.8)	7,840 (10.6)	
	70–79	23,241 (6.7)	20,576 (7.5)	2,665 (3.6)		2,785 (3.8)	2,665 (3.6)	
	80+	6,407 (1.8)	5,444 (2.0)	963 (1.3)		775 (1.0)	963 (1.3)	
Sex	F	151,608 (43.7)	117,830 (43.2)	33,778 (45.8)	0.052	33,403 (45.3)	33,778 (45.8)	0.010
	M	195,124 (56.3)	155,088 (56.8)	40,036 (54.2)		40,411 (54.7)	40,036 (54.2)	
Country of Origin	Africa	11,812 (3.4)	7,840 (2.9)	3,972 (5.4)	0.168	1,971 (2.7)	3,972 (5.4)	0.191
	Asia	36,104 (10.4)	26,782 (9.8)	9,322 (12.6)		6,847 (9.3)	9,322 (12.6)	
	Europe	35,909 (10.4)	28,736 (10.5)	7,173 (9.7)		7,381 (10.0)	7,173 (9.7)	
	N America	2,589 (0.7)	2,046 (0.7)	543 (0.7)		570 (0.8)	543 (0.7)	
	Other	777 (0.2)	646 (0.2)	131 (0.2)		169 (0.2)	131 (0.2)	
	Sweden	251,308 (72.5)	200,720 (73.5)	50,588 (68.5)		55,258 (74.9)	50,588 (68.5)	
	S America	7,506 (2.2)	5,590 (2.0)	1,916 (2.6)		1,433 (1.9)	1,916 (2.6)	
	Unknown	727 (0.2)	558 (0.2)	169 (0.2)		185 (0.3)	169 (0.2)	
Education	Primary	37,358 (10.8)	28,273 (10.4)	9,085 (12.3)	0.087	7,464 (10.1)	9,085 (12.3)	0.100
	Secondary	122,173 (35.2)	94,990 (34.8)	27,183 (36.8)		25,407 (34.4)	27,183 (36.8)	
	Tertiary	186,374 (53.8)	149,016 (54.6)	37,358 (50.6)		40,731 (55.2)	37,358 (50.6)	
	Unknown	827 (0.2)	639 (0.2)	188 (0.3)		212 (0.3)	188 (0.3)	
Charlson Comorbidity Index Score	0	305,620 (88.1)	239,594 (87.8)	66,026 (89.4)	0.054	67,600 (91.6)	66,026 (89.4)	0.073
	1–2	36,866 (10.6)	29,815 (10.9)	7,051 (9.6)		5,654 (7.7)	7,051 (9.6)	
	3–5	2,872 (0.8)	2,379 (0.9)	493 (0.7)		383 (0.5)	493 (0.7)	
	6+	1,374 (0.4)	1,130 (0.4)	244 (0.3)		177 (0.2)	244 (0.3)	
Primary Care Visits in 2019	0	131,725 (38.0)	101,683 (37.3)	30,042 (40.7)	0.087	30,042 (40.7)	30,042 (40.7)	0
	1–2	129,529 (37.4)	102,169 (37.4)	27,360 (37.1)		27,360 (37.1)	27,360 (37.1)	
	3–5	61,637 (17.8)	49,531 (18.1)	12,106 (16.4)		12,106 (16.4)	12,106 (16.4)	
	6+	23,841 (6.9)	19,535 (7.2)	4,306 (5.8)		4,306 (5.8)	4,306 (5.8)	
Outpatient Specialist Care Visits in 2019	0	170,902 (49.3)	132,335 (48.5)	38,567 (52.2)	0.101	38,422 (52.1)	38,567 (52.2)	0.033
	1–2	93,009 (26.8)	73,223 (26.8)	19,786 (26.8)		19,245 (26.1)	19,786 (26.8)	
	3–5	48,596 (14.0)	39,077 (14.3)	9,519 (12.9)		9,580 (13.0)	9,519 (12.9)	
	6+	34,225 (9.9)	28,283 (10.4)	5,942 (8.0)		6,567 (8.9)	5,942 (8.0)	
Hospitalization Visits 2019	0	320,975 (92.6)	251,972 (92.3)	69,003 (93.5)	0.045	69,003 (93.5)	69,003 (93.5)	0
	1–2	23,097 (6.7)	18,795 (6.9)	4,302 (5.8)		4,302 (5.8)	4,302 (5.8)	
	3–5	2,178 (0.6)	1,757 (0.6)	421 (0.6)		421 (0.6)	421 (0.6)	
	6+	482 (0.1)	394 (0.1)	88 (0.1)		88 (0.1)	88 (0.1)	
Hospitalized due to COVID-19 in 2020*	No	344,151 (99.3)	272,918 (100.0)	71,233 (96.5)	0.269	73,814 (100.0)	71,233 (96.5)	0.269
	Yes	2,581 (0.7)	0 (0.0)	2,581 (3.5)		0 (0.0)	2,581 (3.5)	
ICU treatment due to COVID-19 in 2020*	No	346,145 (99.8)	272,918 (100.0)	73,227 (99.2)	0.127	73,814 (100.0)	73,227 (99.2)	0.127
	Yes	587 (0.2)	0 (0.0)	587 (0.8)		0 (0.0)	587 (0.8)	

* Hospitalized 2020 and ICU visit in 2020 refer to whether an individual was treated for COVID-19 in a hospital setting or Intensive Care Unit setting during 2020. Only entries with a main diagnosis of COVID-19 (ICD10 code U07.1 or U07.2) were included.

SMD = standard mean difference.

### Healthcare use in the full cohort

**Figure 2.**
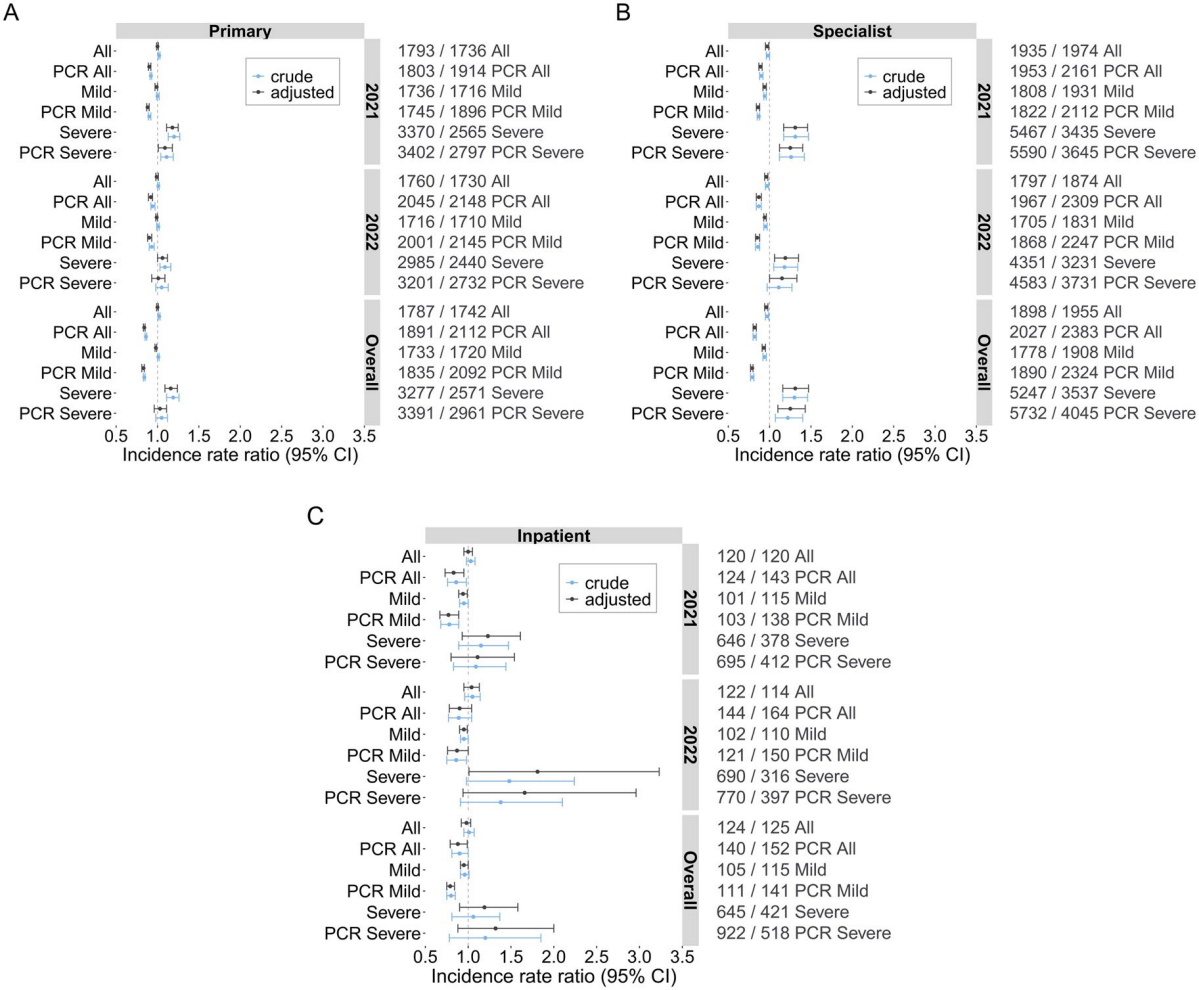
** ** A-C. *Incidence Rate Ratios for the Cohorts*. Primary care (A), Specialist care (B), and inpatient care (C) differences between the sero + and sero- groups during 2021, 2022, and the complete follow-up period (overall). Results for the full cohort, non-hospitalized cohort, and hospitalized cohort are shown here (All, Mild, and Severe, respectively) both with and without PCR censoring during follow-up. Mild IRR values were computed from the non-hospitalized cohort, and Severe IRR values were computed from the hospitalized only cohort. The incidence rates per 1,000 person years are shown on the right for each cohort; the first number showing the incidence for the Sero + group and the second number showing the incidence for the Sero- group.

In total, 1,182/272,918 (0.4%) individuals in the sero- group and 270/73,814 (0.4%) in the sero + group died during follow-up, while 2.2% of the sero- group and 2.1% of the sero + group moved out of Stockholm County. Healthcare use during follow-up for the whole cohort is presented in Supplementary Table 1. Healthcare use during 2021 and 2022 was similar between the sero + and sero- groups (Figure 2(A–C)). For primary care the ratios of difference in incidence rates between sero + and sero- groups, adjusted IRR (95% CI), were 1.00 (0.99–1.01) in 2021, 0.99 (0.98–1.01) in 2022, and 1.00 (0.99–1.01) over the whole follow-up. For specialist outpatient care, the IRRs were 0.97 (0.95–0.99) in 2021, 0.96 (0.94–0.98) in 2022, and 0.96 (0.94–0.98) overall. For inpatient care the IRRs were 1.00 (0.95–1.05) in 2021, 1.04 (0.95–1.13) in 2022, and 0.98 (0.92–1.03) overall. When healthcare visits during the follow-up period were binned as 0, 1–2, 3–5 and 6+, the results were similar (Supplementary Table 2).

**Figure 3.**
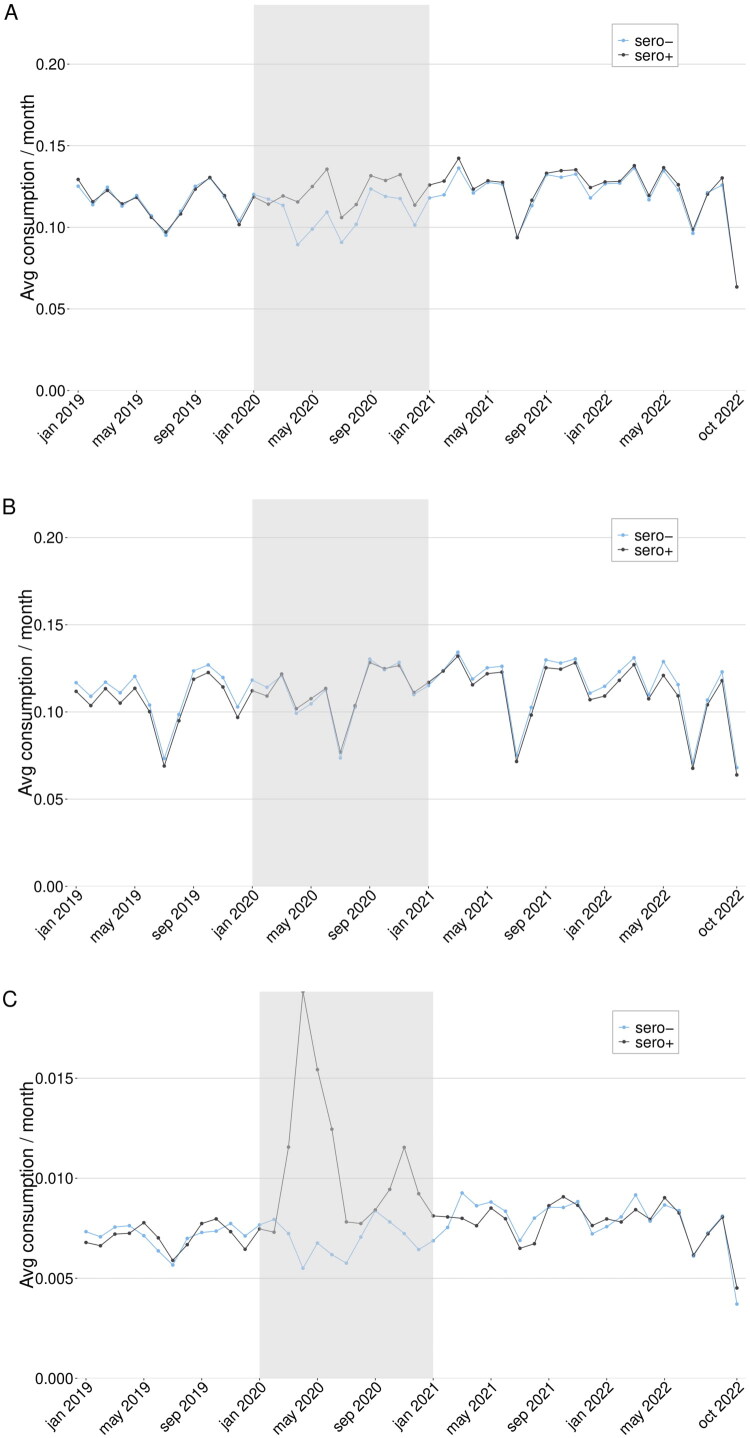
A–C. *Difference-in-differences for the full cohort*. Primary care (A), outpatient specialist care (B), and inpatient care (C) use in the full cohort of sero + and sero- individuals from January 2019 until October 2022. The greyed area represents the time when serology tests were performed as a basis for inclusion in the cohorts. The analysis measured whether an individual used any healthcare service of the given type or not for each month.

Healthcare outcomes were compared by month for the groups (Figure 3(A–C)). Overall, there was an increase in primary care and inpatient care use in the sero + group during 2020, but no substantial differences were observed in 2021 or 2022. The DiD analysis resulted in the following changes in monthly visit rates for the sero + compared to the sero- group (with 0.0 meaning no difference between groups): primary care consumption 0.03 (0.00–0.06) over the whole follow-up period, 0.05 (0.02–0.09) during 2021, and 0.02 (−0.02 − 0.05) during 2022; outpatient specialist care consumption 0.01 (−0.03 − 0.06) overall, 0.02 (−0.04 − 0.08) during 2021, and 0.01 (−0.05 − 0.06) during 2022; inpatient healthcare consumption 0.00 (−0.01 − 0.00) for the whole follow-up period, 0.00 (−0.01 − 0.00) during 2021, and 0.00 (−0.01 − 0.01) during 2022.

The analyses where individuals were censored on positive SARS-CoV-2 PCR tests during follow-up showed differences. In the sero- group, 22,544/73,814 (30.5%) of individuals were censored due to a positive PCR test, while 9,550/73,814 (12.9%) were censored in the sero + group. IRRs for primary care were lower, at 0.90 (0.89–0.92) in 2021, 0.92 (0.89–0.94) in 2022, and with an overall value of 0.84 (0.83–0.85). Specialist care was also lower, at 0.89 (0.87–0.91), 0.87 (0.84–0.9), and 0.82 (0.8–0.84) for 2021, 2022, and overall. Finally, inpatient IRRs were lower at 0.83 (0.73–0.95), 0.9 (0.78–1.04), and 0.88 (0.79–0.99) for 2021, 2022, and overall. The DiD analyses showed minor increases in monthly healthcare use for the sero + group: in primary care the differences were 0.09 (0.05–0.12) for 2021, 0.02 (−0.01 − 0.06) for 2022, and 0.05 (0.02–0.08) overall. Specialist care showed insignificant differences: 0.01 (−0.05 − 0.07) for 2021, −0.03 (−0.09 − 0.03) for 2022, and −0.02 (−0.07 − 0.03) overall. Inpatient monthly use did not show any differences.

### Healthcare use in COVID-19 hospitalized cohort

**Figure 4.**
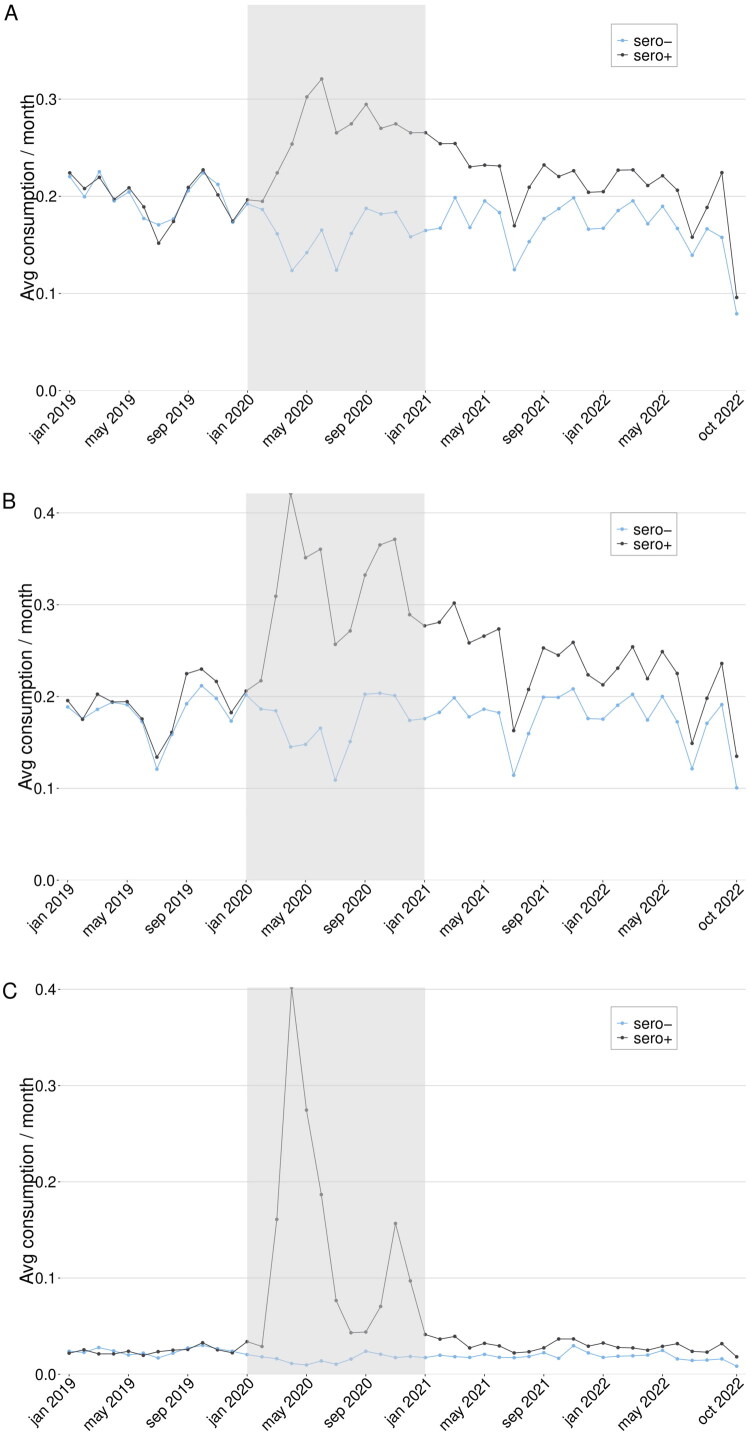
** ** A–C. *Difference-in-differences for the hospitalized cohort*. Primary care (A), outpatient specialist care (B), and inpatient care (C) use from January 2019 until October 2022 in the sero + individuals who were hospitalized for COVID-19 in 2020 and their matched sero- controls. The full cohort consisted of 2,597 matched individuals. The greyed area represents the time when serology tests were performed as a basis for inclusion in the cohorts. The analysis measured whether an individual used any medical service of the given type or not for each month.

We performed stratified analyses of sero + individuals that were hospitalized in 2020 for COVID-19 (*n* = 2,597), and their sero- controls. Contrary to the full cohort, the hospitalized cohort showed substantial increases in primary care, specialist care, and inpatient care during the follow-up period (Figure 2(A–C)). The primary care adjusted IRRs were 1.18 (1.11–1.25), 1.06 (1.00–1.12), and 1.16 (1.09–1.24) for 2021, 2022, and overall; specialist care IRRs 1.31 (1.17–1.46), 1.19 (1.06–1.35), and 1.31 (1.16–1.47), for 2021, 2022, and overall; and inpatient care IRRs 1.23 (0.93–1.61), 1.81 (1.01–3.23), and 1.19 (0.9–1.58), for 2021, 2022, and overall. Compared to the full cohort, the hospitalized cohort showed significant monthly differences between the groups (Figure 4(A–C)). The differences in primary care were 0.83 (0.55–1.1) in 2021, 0.4 (0.15–0.65) in 2022, and 0.61 (0.4–0.83) overall. For specialist care the differences were 1.08 (0.4–1.75), 0.16 (−0.46 − 0.79), and 0.62 (0.07–1.17), for 2021, 2022, and overall. Finally, inpatient care differences were 0.21 (0.11–0.31), 0.13 (0.04–0.22), and 0.17 (0.09–0.25), for 2021, 2022, and overall.

The hospitalized cohort analyses where individuals were censored on a positive SARS-CoV-2 PCR test during follow-up showed subdued results. Of the sero- individuals, 571/2,597 (22%) were censored due to a positive PCR test during follow-up, while 202/2,597 (7.8%) individuals in the sero + group were censored. The primary care IRRs were lower than in the non-PCR-censored analysis, and only the adjusted IRR for 2021 remained significantly elevated at 1.09 (1.01–1.18) (Figure 2(A)). Specialist care showed a smaller reduction from the non-censored version, at adjusted IRRs of 1.25 (1.12–1.40), 1.15 (1.00–1.33), and 1.25 (1.10–1.43) for 2021, 2022, and overall (Figure 2B). For inpatient healthcare use the uncertainty of the estimates was large (Figure 2(C)). The DiD analysis estimates were slightly reduced compared to the non-censored analysis; primary care differences were 0.82 (0.54–1.10) for 2021, 0.37 (0.10–0.63) for 2022, and 0.59 (0.37–0.82) overall. Specialist care differences were .2 (.3 − .1) for 2021, −0.32 (−0.94 − 0.3) for 2022, and 0.31 (−0.23 − 0.86) overall. Inpatient care was 0.18 (0.09–0.28) for 2021, 0.11 (0.02–0.20) for 2022, and 0.15 (0.07–0.23) overall.

### Healthcare use in non-hospitalized cohort

**Figure 5.**
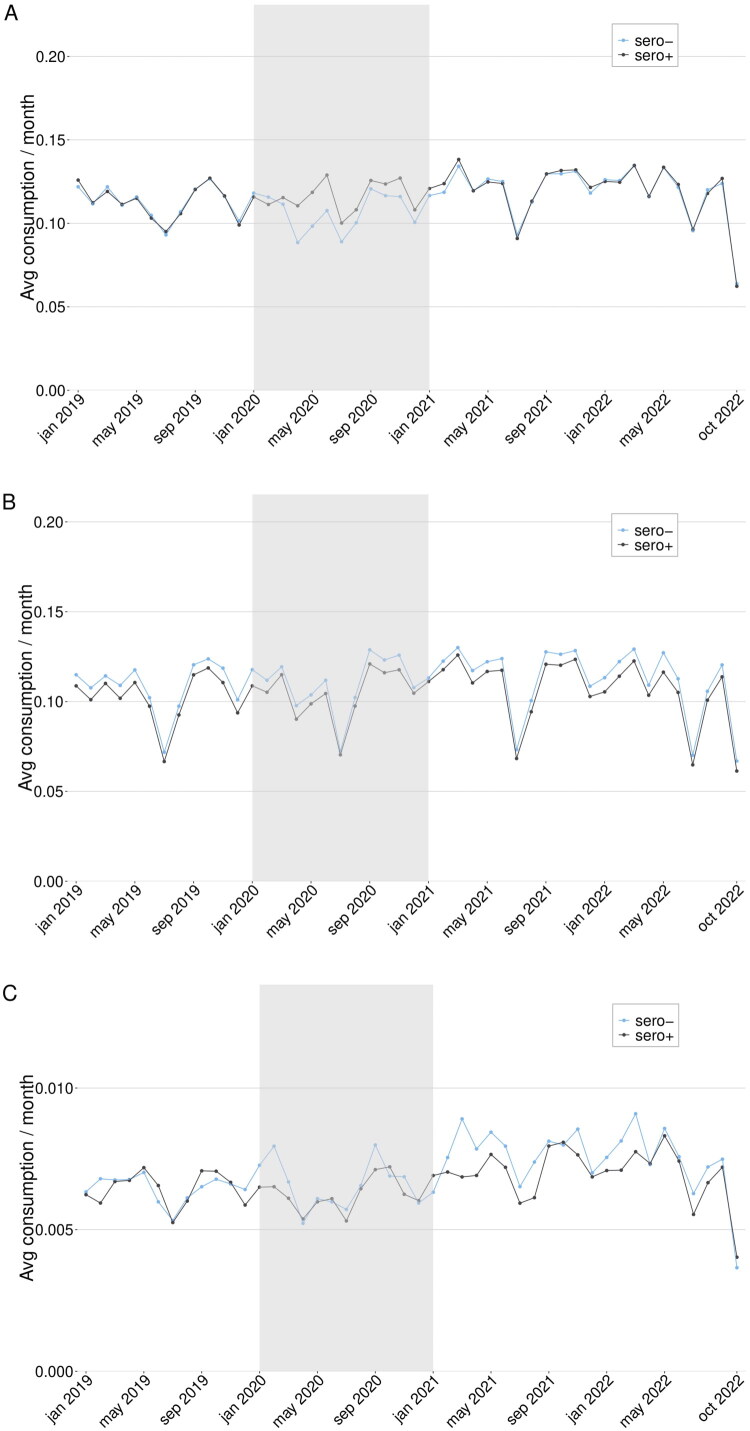
** ** A–C. *Difference-in-differences for the non-hospitalized cohort*. Primary care (A), outpatient specialist care (B), and inpatient care (C) use from January 2019 until October 2022 in the sero + persons without hospitalization for COVID-19 in 2020 and their matched sero- controls. The matched cohort consisted of 71,217 matched individuals. The greyed area represents the time when serology tests were performed as a basis for inclusion in the cohorts. The analysis measured whether an individual used any medical service of the given type or not for each month.

For persons with positive SARS-CoV-2 serology but without hospitalization due to COVID-19 in 2020 (*n* = 71,217), the overall primary care, outpatient specialist visits, and inpatient visits during the follow-up period were reduced: 0.98 (0.97–0.99), 0.93 (0.91–0.95) and 0.95 (0.91–1.00), respectively (Figure 2(A–C)). A similar pattern was observed in the monthly care use (Figure 5(A–C)). The DiD analyses were: primary care use 0.02 (−0.02 − 0.05), 0.00 (−0.03 − 0.03), and 0.01 (−0.02 − 0.04), for 2021, 2022, and overall; specialist care 0.00 (−0.05 − 0.06), 0.00 (−0.04 − 0.05), and 0.00 (−0.04 − 0.05), for 2021, 2022, and the whole follow-up period; and for inpatient care all estimates were −0.01 (−0.01 − 0.00).

The analyses where individuals were censored on a positive SARS-CoV-2 PCR test during follow-up resulted in reduced IRRs (Figure 2(A–C)), with 21,996/71,217 (30.9%) sero- individuals censored and 9,348/71,217 (13.1%) sero + individuals censored. Primary care IRRs were at 0.88 (0.87–0.90), 0.9 (0.88–0.93), and 0.83 (0.81–0.84) for 2021, 2022, and overall. Specialist care IRRs were 0.86 (0.84–0.88), 0.85 (0.83–0.88), and 0.79 (0.77–0.80) for 2021, 2022, and overall. Inpatient healthcare was 0.77 (0.67–0.89) for 2021, 0.87 (0.76–1.00) for 2022, and 0.79 (0.75–0.84) overall. The DiD estimates for the non-hospitalized sero + cohort were for primary care 0.06 (0.02–0.09) for 2021, 0.02 (−0.02 − 0.05) for 2022, and 0.03 (0.00–0.06) overall. Specialist care estimates were 0 (−0.06 − 0.05) for 2021, −0.02 (−0.07 − 0.03) for 2022, and −0.03 (−0.07 − 0.03) overall. All inpatient estimates were −0.01 (−0.02 − 0.00).

### Sensitivity analyses

Both sensitivity analyses, restricting the cohort to serology test performed only within the last month before the start of follow-up, and the analysis where a positive PCR test during 2020 was not used as an exclusion criterion for the sero- group, showed similar results as the main analyses (Supplementary Figure 2(A–C)).

## Discussion

In this population-based study, using SARS-CoV-2 serology as a measure of exposure during 2020, there was no increase in long-term healthcare use during 2021 or 2022 in individuals with a SARS-CoV-2 infection that were not hospitalized due to COVID-19 in 2020. We did observe an increased long-term healthcare consumption in serology-positive individuals who had been hospitalized for COVID-19.

This prevailing increase in the hospitalized/severe infection category is consistent with other studies [23,32,33], but the incidence rates we found in our study population were on the lower end when compared to studies exploring COVID-19 specifically [3–5]. This is expected given the generalized healthcare outcomes we measured; the differences between population level and severe infection IRRs do however suggest that the healthcare burden on the population level beyond severe COVID-19-specific sequelae is limited. This finding has been reported in other populations, among them a multicenter healthcare study in Israel [34] that found mild cases of COVID-19 in the 299,870 infected individuals to hold few long-term healthcare consequences, and a study on 242,712 previously infected individuals in England that observed only 5.2% of previously infected still self-reporting on-going symptoms 52 weeks or more after the acute infection [35]. The English study also reported that comorbidities and the wild-type strain were the most likely predictors of long-term self-reported health issues, approximating our setup and results to some degree. The Israeli study reported no increased long-term risk for hospital admissions.

Censoring the population on a positive SARS-CoV-2 PCR test during the follow-up period resulted in lower IRRs, most notably the analyses restricted to sero + individuals with mild COVID-19 during 2020. Sero- individuals without immunity will have had a higher rate of SARS-CoV-2 infection in 2021/22, with the possible result of higher healthcare use due to these infections. Censoring these individuals resulted in a sero- group that remained uninfected according to records. Second, as vaccinations became available in Sweden 2020-12-27, using positive PCR tests for censoring during the follow-up period was more likely to censor unvaccinated individuals and individuals that were more likely to experience a severe acute phase of the illness [36], resulting in more severe post-acute sequelae [37]. The number of individuals censored was consistent across the different cohorts, at slightly below 3:1 between sero- and sero + groups. The use of NKCOV and SmiNet as sources for PCR-based censoring largely covers the entire population of Stockholm County as reporting communicable diseases to SmiNet is mandatory. This is the most comprehensive source of COVID-19 cases available in Sweden, though it does not cover self-tested or self-treated infections, a phenomenon presumably more common towards the tail-end of the follow-up when home-based antigen testing was more commonly employed.

The increase in specialist care consumption did not persist in the DiD analysis. This is likely a result of how specialist care patterns appear in Sweden: those that do employ these services tend to use them frequently, a factor which is diminished in the DiD comparison.

The COVID-19 pandemic began in 2020, with new variants developing and spreading over the next years and hospitalization risk decreasing towards the end. Omicron, the last variant of the pandemic period, had the lowest risk of hospitalization [38]. Consequences from infections after the pandemic can thus be difficult to compare against infections during 2020. Given the relation between severity of infection and convalescence period/PACS [23], future infections are also likely to have a reduced impact on healthcare use. As such, infections from 2020 are likely to show an upper limit of the possible consequences compared to future effects of SARS-CoV-2, making them a suitable data source for informing public health and surveillance policy.

Since the discovery of PACS many concerns have been raised regarding the long-term burden of the COVID-19 pandemic. Our findings show that such a burden has not materialized, nor is it likely to ever do so, with regards to healthcare use volume.

### Strengths

While PCR testing finds ongoing infections, serology testing detects the previously infected during a larger time window [13]. This helps with classifying asymptomatic cases unlikely to be detected by symptom-initiated healthcare services. General serology testing in Stockholm began in June; the distribution for the last test taken in 2020 matching variable for our study population peaks in June-July and November-December (Supplementary Figure 1), suggesting that we capture information about the initial waves. With access to serology data, PCR data, and hospital records, we were able to filter out gaps in the serology data for inclusion in the study, approximating serology coverage and infection events. The sensitivity analyses (Supplementary Figure 2(A–C)) show similar IRRs to the matched cohort indicating that the results are robust with regards to outliers. The analysis for severe COVID-19, requiring hospitalization in 2020, shows the increased healthcare use also reported in other studies, indicating that the data is consistent with other clinical data-sourced studies. The hospitalized cohort consisted of 2,597 individuals, which held enough power to show a substantial increase in healthcare use. This suggests that the study population design is more than sufficient to show the effects of COVID-19 on healthcare use.

### Limitations

The inclusion criteria for the study population were based on evaluating antibody response towards SARS-CoV-2, signifying previous infection. Variability in serology is commonly higher than for PCR [39]. Sensitivity analyses restricted to serologies performed close to the start of follow-up showed consistent results, indicating that misclassification due to waning antibody levels is not a major issue. It is important to note that the outcome was the use of healthcare services, not well-being, meaning that health issues not leading to healthcare use are excluded in this analysis. While there are many studies available on perceived and self-reported sequelae of PCC/PACS/PASC, these symptoms would not necessarily translate into additional healthcare use. Our study population had minor differences to the Stockholm County population (slightly more likely to be middle-aged, middle class, and university educated (Supplementary Figure 3)).

## Conclusions

The results indicate that there is no substantially increased long-term healthcare burden in the population of subjects who had SARS-CoV-2 infection in 2020, as determined by serology testing. Consequently, healthcare systems do not need to adjust for an expected increase in volume of healthcare use by the population due to COVID-19 sequelae.

## Acknowledgements

We would like to acknowledge Pouran Almstedt for her contributions to the development of the National Quality Registry for SARS-CoV2 (NKCOV). We would like to acknowledge Sandra Muschiol for her assistance with lab protocols and analysis platform descriptions.

## Contributors

Nicholas Baltzer had full access to all the data in the study and takes responsibility for the integrity of the data and the accuracy of the data analysis. All authors have read and approved the manuscript.

Conceptualization: Nicholas Baltzer, Joakim Dillner, Pontus Nauclér

Data curation: Nicholas Baltzer, Sara Nordqvist-Kleppe

Formal analysis: Nicholas Baltzer

Funding acquisition: Pär Sparén, Pontus Nauclér

Investigation: Nicholas Baltzer

Methodology: Nicholas Baltzer, Pontus Nauclér, Pontus Hedberg

Project administration: Nicholas Baltzer

Resources: Nicholas Baltzer, Pontus Nauclér

Software: Nicholas Baltzer

Supervision: Pontus Nauclér

Validation: Nicholas Baltzer, Pontus Hedberg, Sara Nordqvist Kleppe, Joakim Dillner, Anders Sönnerborg, Jan Albert, Kristoffer Strålin, Pär Sparén, Pontus Nauclér

Visualization: Nicholas Baltzer

Writing original draft: Nicholas Baltzer

Writing – review & editing: Nicholas Baltzer, Pontus Hedberg, Sara Nordqvist Kleppe, Joakim Dillner, Anders Sönnerborg, Jan Albert, Kristoffer Strålin, Pär Sparén, Pontus Nauclér.

## Supplementary Material

Supplemental Material

## Data Availability

The individual participant data underlying this article were subject to ethical approval and cannot be shared publicly. Data from the deidentified administrative health registry are not freely available due to protection of the personal integrity of the participants.
