# Supplementary material for: Long-term healthcare use of COVID-19 cases in 2020: a two-year follow-up in Stockholm, Sweden
Source: Ann Med. 2025 Oct 31;57(1):2580077. doi: 10.1080/07853890.2025.2580077 (PMC12581745; doi:10.1080/07853890.2025.2580077)
Supplement: Supplemental Material [file IANN_A_2580077_SM1394.zip › suppl_data/manuscript_sero2020_AoM_captions.docx]

Captions

Supplementary Figure 1

**Supplementary Figure 1**. Histogram with side-by-side bars of the last test date in 2020 for the study population by serology test result (count on left-side y axis). Each bar represents a week. The dark red line corresponds to overall COVID-19 hospitalizations in Stockholm County during the same time period (numbers of admissions on the right y axis).

Supplementary Figure 2 (panel)

**Supplementary Figure 2A-2C**. Sensitivity analysis Incidence Rate Ratios (IRR), both crude (blue) and adjusted (black), for primary care (A), specialist care (B), and inpatient care (C), during 2021, 2022, and over the complete follow-up period (overall). The full sero+ cohort is included for reference. The incidence rates per 1,000 person years are shown on the right for each cohort; the first number showing the incidence for the sero+ group and the second number showing the incidence for the sero- group. The ‘No PCR Exclusion’ bars refer to the sensitivity analysis where exclusion from the sero- group due to a positive PCR test in 2020 was omitted. ‘The Last Month Only’ bars refer to the sensitivity analysis where inclusion into the cohort additionally required that the sample date for any serology test was taken no earlier than 2020-11-25, i.e. the last month of the inclusion period.

Supplementary Figure 3 (panel)

**Supplementary Figure 3**. Summary of generalization from the study population onto the 18+ Stockholm general public, showing population densities for birth date, categorized education level and disposable income. The study population is slightly more likely to be middle-aged, slightly more likely to have a master’s degree or equivalent, and has on average a slightly higher salary.
