# Supplementary material for: Long-term healthcare use of COVID-19 cases in 2020: a two-year follow-up in Stockholm, Sweden
Source: Ann Med. 2025 Oct 31;57(1):2580077. doi: 10.1080/07853890.2025.2580077 (PMC12581745; doi:10.1080/07853890.2025.2580077)
Supplement: Supplemental Material [file IANN_A_2580077_SM1394.zip › suppl_data/manuscript_sero2020_appendix_AoM.docx]

**Appendix**

## **SARS-CoV-2 serology**

Results from SARS-CoV-2 serology obtained between 2020-01-31 and 2020-12-26 were included in the analyses. Several different analysis platforms were used during this time period. An a priori decision was made to exclude tests performed on analysis platforms with fewer than 1,000 records. Results from five different commercial SARS-CoV-2 serology assays and one in-house assay met these criteria and are presented below. The Abbott platforms were used at Synlab Central Laboratory, Täby Sweden and the remaining platforms were used at Clinical Microbiology, Karolinska University Hospital, Stockholm, Sweden. Both laboratories are accredited.

*SARS-CoV-2 Ak, Cobas Pro (Roche)*

We included 222,593 test results generated between 2020-06-26 and 2020-12-26 at Clinical Microbiology, Karolinska University Hospital, Stockholm Sweden on the Roche Elecsys Anti-SARS-CoV-2 assay platform. The assay measures total antibody against nucleocapsid of SARS-CoV-2 and was used according to manufacturer’s instructions on a Cobas Pro instrument.

*SARS-CoV-2 IgG, MB Architect (Abbott)*

We included 33,385 test results generated between 2020-06-09 and 2020-12-23 on the Abbott MB Architect platform. The assay reports the sample/control index ratio measuring IgG antibodies against nucleocapsid protein and was used according to manufacturer´s instructions on an MB Architect analyzer.

*SARS-CoV-2 IgG, MB Architect II (Abbott)*

We included 43,198 test results generated between 2020-05-27 and 2020-12-23 on the Abbott MB Architect II platform. The assay reports the sample/control index ratio measuring IgG antibodies against nucleocapsid protein and was used according to manufacturer´s instructions on an MB Architect analyzer.

*SARS-CoV-2 IgG-Ak, iFlash (YHLO)*

We included 35,129 test results generated between 2020-05-04 and 2020-10-16 on the SARS-CoV-2 IgG-Ak, iFlash (YHLO) platform (cat no. YHL-C86095G). The assay detects IgG against both the nucleocapsid and spike protein and was used according to manufacturer´s instructions on an iFlash 1800 analyzer.

*SARS-CoV-2 IgG-Ak, Liaison XL (Diasorin)*

We included 16,518 test results generated between 2020-06-17 and 2020-08-10 on the LIAISON SARS-CoV-2 S1/S2 IgG (cat. No. 311450) platform. The assay detects IgG against the S1 and S2 domain of the spike protein and was used according to manufacturer´s instructions on a Liaison XL analyzer.

*SARS-CoV-2 IgG-Ak (in-house)*

We included 2,082 test results generated between 2020-04-21 and 2020-06-12 by an in-house ELISA assay that detects IgG against the nucleocapsid protein of SARS-CoV-2. The assay was developed by Matti Sällberg and Gustaf Ahlén, Department of Laboratory Medicine, Karolinska Institutet, who kindly provided recombinant nucleocapsid protein (Genscript). In brief, 96-well ELISA plates (Nunc MaxiSorp) were coated with nucleocapsid protein (1 µg/ml) in 50 mM sodium carbonate buffer pH 9.6 for ~15 h at 4°C. Plates were washed three times with PBS-Tween-20 (0.05%) and blocked using PBS containing 1% BSA and 2% goat serum (Sigma) for 1 h at 37°C. Plates were then washed as before and incubated with human serum samples for 1 h at 37°C. Samples were diluted at 1:600 in PBS-1% BSA-2% goat serum. After washing as before, secondary HRP-conjugated goat anti-human antibodies (Sigma) diluted at 1:40,000 in PBS-1% BSA-2% goat serum was added to the plates and incubated for 1 h at 37°C. Plates were washed a final time before development with TMB (Sigma) kept at 4°C. The reaction was stopped using 2M sulphuric acid. If not stated otherwise, the assay was carried out automated using a TECAN Freedom Evolyzer 2 and optical density values were measured at 450 nm. The cut-of value was based on the 98th percentile. The specificity of the assay was determined to be 98.8% (247/250) by testing samples from healthy blood donors collected during 2019 before the outbreak of SARS CoV-2. The sensitivity was determined to be 79.49% (31/39) for samples obtained ≥ 14 days after onset of symptoms.
