# Supplementary material for: Long-term healthcare use of COVID-19 cases in 2020: a two-year follow-up in Stockholm, Sweden
Source: Ann Med. 2025 Oct 31;57(1):2580077. doi: 10.1080/07853890.2025.2580077 (PMC12581745; doi:10.1080/07853890.2025.2580077)
Supplement: Supplemental Material [file IANN_A_2580077_SM1394.zip › suppl_data/s_table1_consumption_during.docx]

Supplementary Table 1 Healthcare consumption during the follow-up period per person-years at risk in the whole cohort.

|  |  | **sero-** | **sero+** | **Crude IRR (95% CI)** | **Adjusted** IRR (95% CI)** |
| --- | --- | --- | --- | --- | --- |
| **N** | ·· | 272,918 | 73,814 | ·· | ·· |
| **Total patient-years of risk** | ·· | 482,595 | 130,680 | ·· | ·· |
| **Primary Care Visits*** | 0 | 62,151 (22.8) | 17,916 (24.3) | 1.06 (1.05, 1.08) | 1.01 (1.00, 1.02) |
|  | 1-2 | 88,739 (32.5) | 24,516 (33.2) | 1.02 (1.01, 1.04) | 1.01 (0.99, 1.02) |
|  | 3-5 | 69,501 (25.5) | 18,464 (25.0) | 0.98 (0.97, 1.00) | 1.01 (0.99, 1.02) |
|  | 6+ | 52,527 (19.2) | 12,918 (17.5) | 0.91 (0.89, 0.93) | 0.94 (0.93, 0.96) |
| **Primary Care Visits in 2021** | 0 | 99,046 (36.3) | 28,061 (38.0) | 1.05 (1.03, 1.06) | 1.01 (0.99, 1.02) |
|  | 1-2 | 102,533 (37.6) | 27,716 (37.5) | 1.0 (0.99, 1.01) | 1.01 (1.00, 1.02) |
|  | 3-5 | 50,820 (18.6) | 12,923 (17.5) | 0.94 (0.92, 0.96) | 0.98 (0.96, 0.99) |
|  | 6+ | 20,519 (7.5) | 5,114 (6.9) | 0.91 (0.89, 0.94) | 0.94 (0.91, 0.97) |
| **Primary Care Visits in 2022** | 0 | 117,001 (42.9) | 33,306 (45.1) | 1.05 (1.04, 1.06) | 1.01 (1.00, 1.02) |
|  | 1-2 | 101,922 (37.3) | 27,046 (36.6) | 0.98 (0.97, 0.99) | 1.00 (0.99, 0.91) |
|  | 3-5 | 41,532 (15.2) | 10,423 (14.1) | 0.93 (0.91, 0.95) | 0.96 (0.94, 0.98) |
|  | 6+ | 12,463 (4.6) | 3,039 (4.1) | 0.90 (0.87, 0.94) | 0.93 (0.89, 0.96) |
| **Specialist Care Visits *** | 0 | 98,258 (36.0) | 28,770 (39.0) | 1.08 (1.07, 1.10) | 1.01 (1.00, 1.02) |
|  | 1-2 | 68,123 (25.0) | 19,031 (25.8) | 1.03 (1.02, 1.05) | 1.03 (1.01, 1.04) |
|  | 3-5 | 47,711 (17.5) | 12,574 (17.0) | 0.98 (0.96, 1.00) | 1.01 (0.99, 1.03) |
|  | 6+ | 58,826 (21.6) | 13,439 (18.2) | 0.84 (0.83, 0.86) | 0.92 (0.91, 0.94) |
| **Specialist Care Visits in 2021** | 0 | 129,957 (47.6) | 37,500 (50.8) | 1.07 (1.05, 1.08) | 1.01 (1.01, 1.02) |
|  | 1-2 | 72,822 (26.7) | 19,410 (26.3) | 0.99 (0.97, 1.00) | 1.00 (0.99, 1.02) |
|  | 3-5 | 39,107 (14.3) | 10,006 (13.6) | 0.94 (0.92, 0.97) | 1.00 (0.98, 1.02) |
|  | 6+ | 31,032 (11.4) | 6,898 (9.3) | 0.82 (0.90, 0.84) | 0.90 (0.88, 0.92) |
| **Specialist Care Visits in 2022** | 0 | 147,271 (54.0) | 42,493 (57.6) | 1.07 (1.05, 1.08) | 1.02 (1.01, 1.02) |
|  | 1-2 | 70,394 (25.8) | 18,751 (25.4) | 0.98 (0.97, 1.00) | 1.01 (1.00, 1.03) |
|  | 3-5 | 33,913 (12.4) | 8,098 (11.0) | 0.88 (0.86, 0.90) | 0.95 (0.93, 0.97) |
|  | 6+ | 21,340 (7.8) | 4,472 (6.1) | 0.77 (0.75, 0.8) | 0.86 (0.83, 0.89) |
| **Inpatient Care Visits *** | 0 | 236,925 (86.8) | 65,205 (88.3) | 1.02 (101, 1.03) | 1.01 (1.00, 1.01) |
|  | 1-2 | 30,423 (11.1) | 7,495 (10.2) | 0.91 (0.89, 0.94) | 0.95 (0.93, 0.97) |
|  | 3-5 | 4,158 (1.5) | 844 (1.1) | 0.75 (0.70, 0.81) | 0.89 (0.82, 0.95) |
|  | 6+ | 1,412 (0.5) | 270 (0.4) | 0.72 (0.64, 0.80) | 0.91 (0.81, 1.02) |
| **Inpatient Care Visits in 2021** | 0 | 250,404 (91.8) | 68,594 (92.9) | 1.01 (1.00, 1.02) | 1.00 (1.00, 1.01) |
|  | 1-2 | 19,745 (7.2) | 4,669 (6.3) | 0.88 (0.85, 0.90) | 0.92 (0.89, 0.95) |
|  | 3-5 | 2,174 (0.8) | 440 (0.6) | 0.75 (0.68, 0.83) | 0.89 (0.81, 0.99) |
|  | 6+ | 595 (0.2) | 111 (0.2) | 0.70 (0.59, 0.82) | 0.90 (0.76, 1.06) |
| **Inpatient Care Visits in 2022** | 0 | 255,361 (93.6) | 69,561 (94.2) | 1.01 (1.00, 1.01) | 1.00 (1.00, 1.00) |
|  | 1-2 | 15,424 (5.7) | 3,831 (5.2) | 0.92 (0.88, 0.95) | 0.98 (0.95, 1.02) |
|  | 3-5 | 1,693 (0.6) | 353 (0.5) | 0.77 (0.68, 0.86) | 0.91 (0.81, 1.02) |
|  | 6+ | 440 (0.2) | 69 (0.1) | 0.65 (0.52, 0.82) | 0.81 (0.65, 1.02) |

** Adjusted for age, sex, Charlson comorbidity index, education level, country of origin, primary care use in 2019 and inpatient care use in 2019.
