# Supplementary material for: Long-term healthcare use of COVID-19 cases in 2020: a two-year follow-up in Stockholm, Sweden
Source: Ann Med. 2025 Oct 31;57(1):2580077. doi: 10.1080/07853890.2025.2580077 (PMC12581745; doi:10.1080/07853890.2025.2580077)
Supplement: Supplemental Material [file IANN_A_2580077_SM1394.zip › suppl_data/s_table2_consumption_during_person_years.docx]

Supplementary Table 2 Healthcare consumption during the follow-up period per person-years at risk in the matched cohorts.

|  |  | **Matched sero-** | **Matched sero+** | **Crude IRR (95% CI)** | **Adjusted ** IRR (95% CI)** |
| --- | --- | --- | --- | --- | --- |
| **N** | ·· | 73,814 | 73,814 | ·· | ·· |
| **Total patient-years of risk** | ·· | 130,288 | 130,680 | ·· | ·· |
| **Primary Care Visits*** | 0 | 18,269 (24.8) | 17,916 (24.3) | 0.98 (0.96, 1.00) | 0.99 (0.98, 1.01) |
|  | 1-2 | 24,946 (33.8) | 24,516 (33.2) | 0.98 (0.96, 1.00) | 0.99 (0.98, 1.01) |
|  | 3-5 | 17,958 (24.3) | 18,464 (25.0) | 1.02 (1.01, 1.05) | 1.02 (1.00, 1.04) |
|  | 6+ | 12,641 (17.1) | 12,918 (17.5) | 1.02 (0.99, 1.04)) | 0.97 (0.95, 0.99) |
| **Primary Care Visits in 2021** | 0 | 28,570 (38.7) | 28,061 (38.0) | 0.98 (0.96, 0.99) | 0.99 (0.98, 1.01) |
|  | 1-2 | 27,803 (37.7) | 27,716 (37.5) | 1.00 (0.98, 1.01) | 1.00 (0.99, 1.01) |
|  | 3-5 | 12,688 (17.2) | 12,923 (17.5) | 1.02 (0.99, 1.04) | 1.00 (0.97, 1.02) |
|  | 6+ | 4,753 (6.4) | 5,114 (6.9) | 1.06 (1.02, 1.10) | 1.00 (0.96, 1.03) |
| **Primary Care Visits in 2022** | 0 | 33,676 (45.6) | 33,306 (45.1) | 0.99 (0.97, 1.00) | 1.00 (0.99, 1.01) |
|  | 1-2 | 26,951 (36.5) | 27,046 (36.6) | 1.00 (0.98, 1.02) | 1.00 (0.99, 1.02) |
|  | 3-5 | 10,253 (13.9) | 10,423 (14.1) | 1.01 (0.99, 1.04) | 0.98 (0.96, 1.01) |
|  | 6+ | 2,934 (4.0) | 3,039 (4.1) | 1.03 (0.98, 1.09) | 0.97 (0.92, 1.01) |
| **Specialist Care Visits *** | 0 | 28,854 (39.1) | 28,770 (39.0) | 0.99 (0.98, 1.01) | 1.00 (0.99, 1.02) |
|  | 1-2 | 18,835 (25.5) | 19,031 (25.8) | 1.01 (0.99, 1.03) | 1.01 (1.00, 1.03) |
|  | 3-5 | 12,133 (16.4) | 12,574 (17.0) | 1.03 (1.01, 1.06) | 1.03 (1.01, 1.06) |
|  | 6+ | 13,992 (19.0) | 13,439 (18.2) | 0.96 (0.93, 0.98) | 0.94 (0.92, 0.96) |
| **Specialist Care Visits in 2021** | 0 | 37,577 (50.9) | 37,500 (50.8) | 0.99 (0.98, 1.01) | 1.00 (0.99, 1.01) |
|  | 1-2 | 19,282 (26.1) | 19,410 (26.3) | 1.01 (0.99, 1.03) | 1.01 (0.99, 1.02) |
|  | 3-5 | 9,571 (13.0) | 10,006 (13.6) | 1.04 (1.01, 1.07) | 1.03 (1.01, 1.06) |
|  | 6+ | 7,384 (10.0) | 6,898 (9.3) | 0.93 (0.90, 0.96) | 0.92 (0.89, 0.95) |
| **Specialist Care Visits in 2022** | 0 | 42,296 (57.3) | 42,493 (57.6) | 1.00 (0.99, 1.02) | 1.01 (1.00, 1.02) |
|  | 1-2 | 18,364 (24.9) | 18,751 (25.4) | 1.02 (1.00, 1.04) | 1.02 (1.00, 1.03) |
|  | 3-5 | 8,188 (11.1) | 8,098 (11.0) | 0.99 (0.96, 1.02) | 0.98 (0.95, 1.01) |
|  | 6+ | 4,966 (6.7) | 4,472 (6.1) | 0.90 (0.86, 0.94) | 0.88 (0.85, 0.92) |
| **Inpatient Care Visits *** | 0 | 65,081 (88.2) | 65,205 (88.3) | 1.00 (0.99, 1.01) | 1.00 (1.00, 1.00) |
|  | 1-2 | 7,621 (10.3) | 7,495 (10.2) | 0.98 (0.95, 1.01) | 0.98 (0.95, 1.01) |
|  | 3-5 | 821 (1.1) | 844 (1.1) | 1.03 (0.93, 1.13) | 0.97 (0.88, 1.07) |
|  | 6+ | 291 (0.4) | 270 (0.4) | 0.98 (0.85, 1.13) | 0.88 (0.76, 1.01) |
| **Inpatient Care Visits in 2021** | 0 | 68,418 (92.7) | 68,594 (92.9) | 1.00 (0.99, 1.01) | 1.00 (1.00, 1.00) |
|  | 1-2 | 4,837 (6.6) | 4,669 (6.3) | 0.96 (0.92, 1.00) | 0.96 (0.92, 1.00) |
|  | 3-5 | 426 (0.6) | 440 (0.6) | 1.03 (0.91, 1.18) | 0.96 (0.84, 1.01) |
|  | 6+ | 133 (0.2) | 111 (0.2) | 0.95 (0.77, 1.17) | 0.85 (0.68, 1.05) |
| **Inpatient Care Visits in 2022** | 0 | 69,660 (94.4) | 69,561 (94.2) | 1.00 (0.99, 1.01) | 1 (0.99, 1.00) |
|  | 1-2 | 3,728 (5.1) | 3,831 (5.2) | 1.02 (0.98, 1.07) | 1.02 (0.98, 1.07) |
|  | 3-5 | 345 (0.5) | 353 (0.5) | 1.02 (0.88, 1.18) | 0.94 (0.81, 1.10) |
|  | 6+ | 81 (0.1) | 69 (0.1) | 1.01 (0.75, 1.35) | 0.90 (0.67, 1.20) |

*Healthcare consumption during whole follow-up period

** Adjusted for age, sex, Charlson comorbidity index, education level, country of origin, primary care visits in 2019, and inpatient care visits in 2019.
