# Supplementary figures and images for: Long-term healthcare use of COVID-19 cases in 2020: a two-year follow-up in Stockholm, Sweden
Source: Ann Med. 2025 Oct 31;57(1):2580077. doi: 10.1080/07853890.2025.2580077 (PMC12581745; doi:10.1080/07853890.2025.2580077)

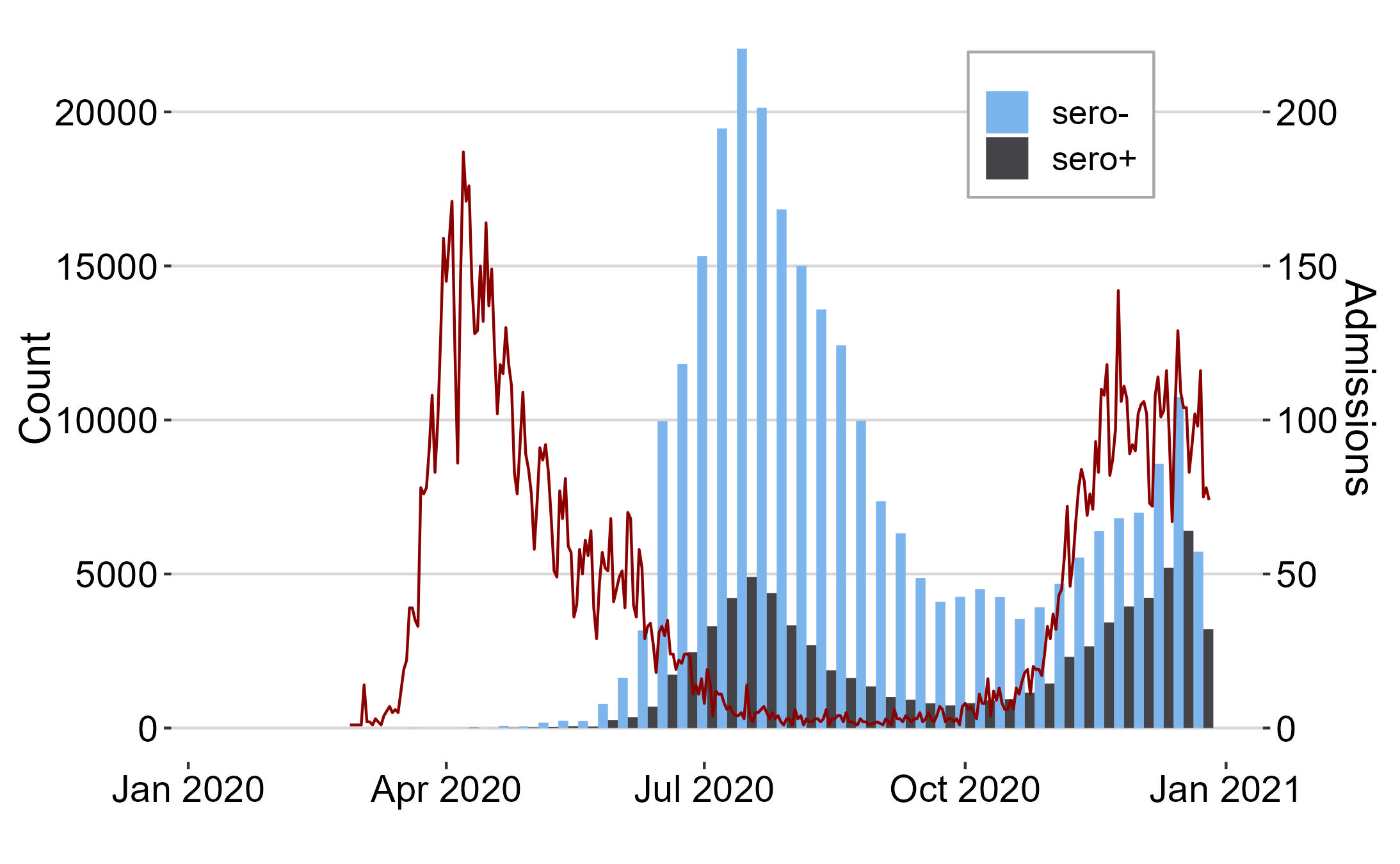

Supplement: Supplemental Material [file IANN_A_2580077_SM1394.zip › suppl_data/fig_s_1.jpg]

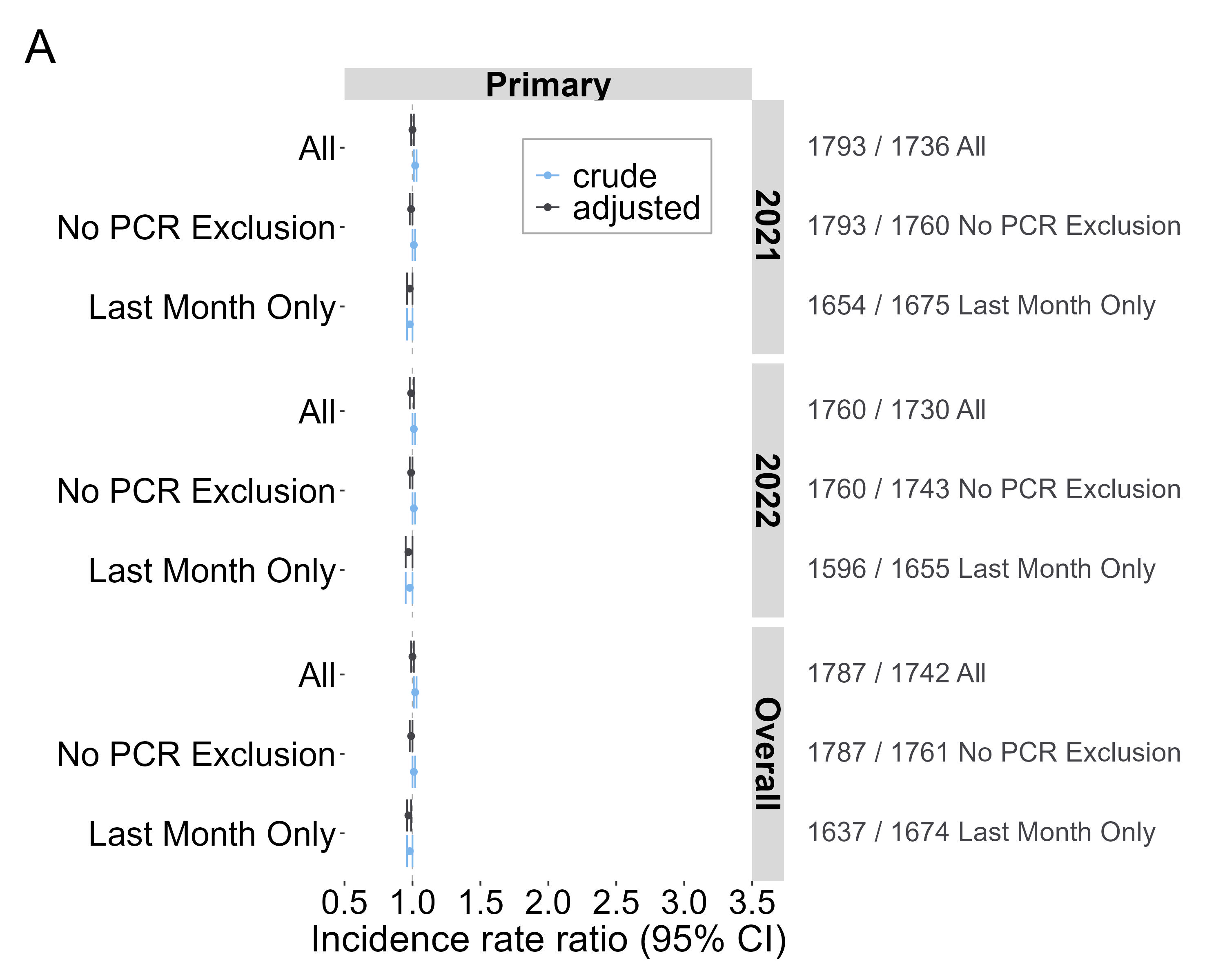

Supplement: Supplemental Material [file IANN_A_2580077_SM1394.zip › suppl_data/fig_s_2A.jpg]

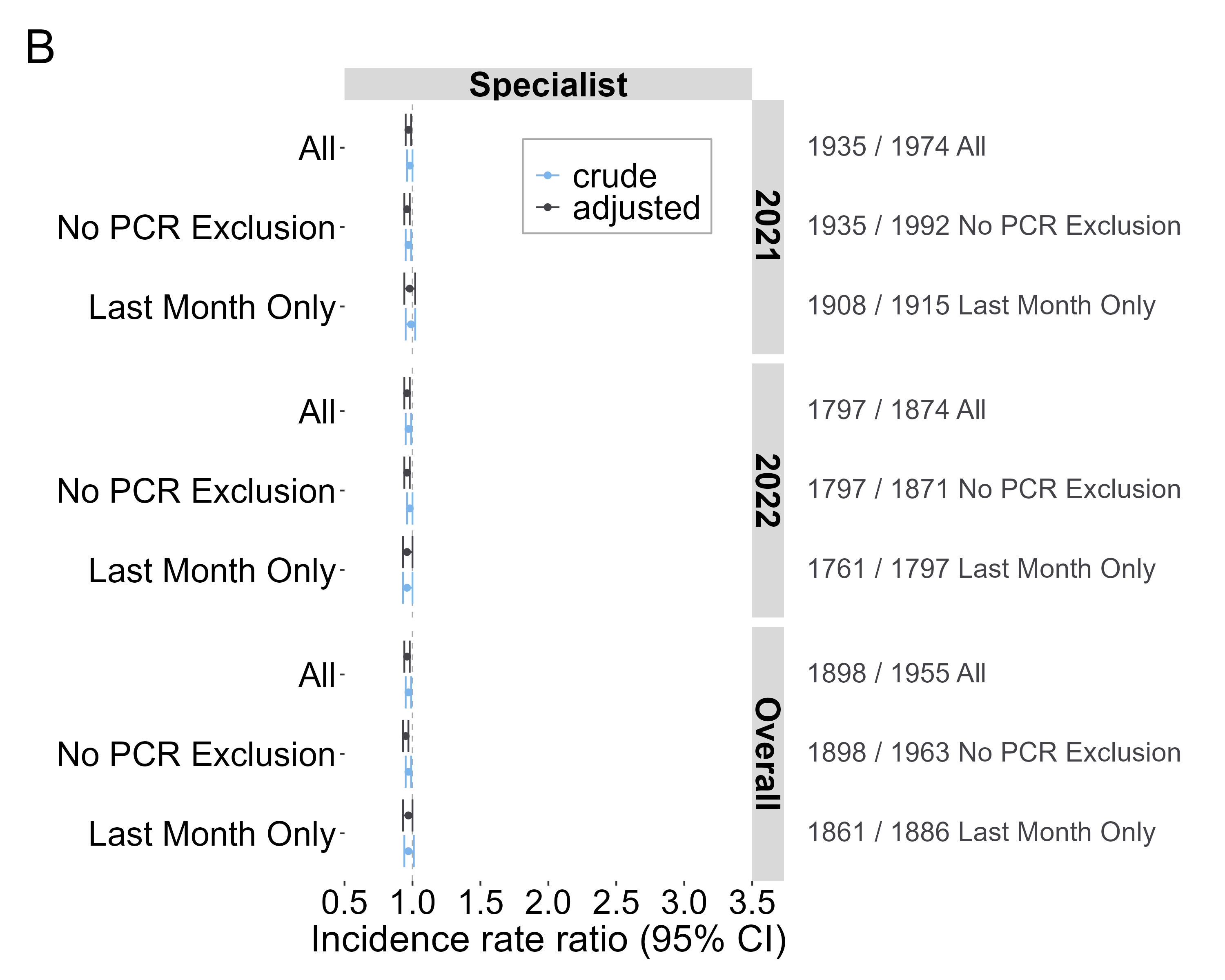

Supplement: Supplemental Material [file IANN_A_2580077_SM1394.zip › suppl_data/fig_s_2B.jpg]

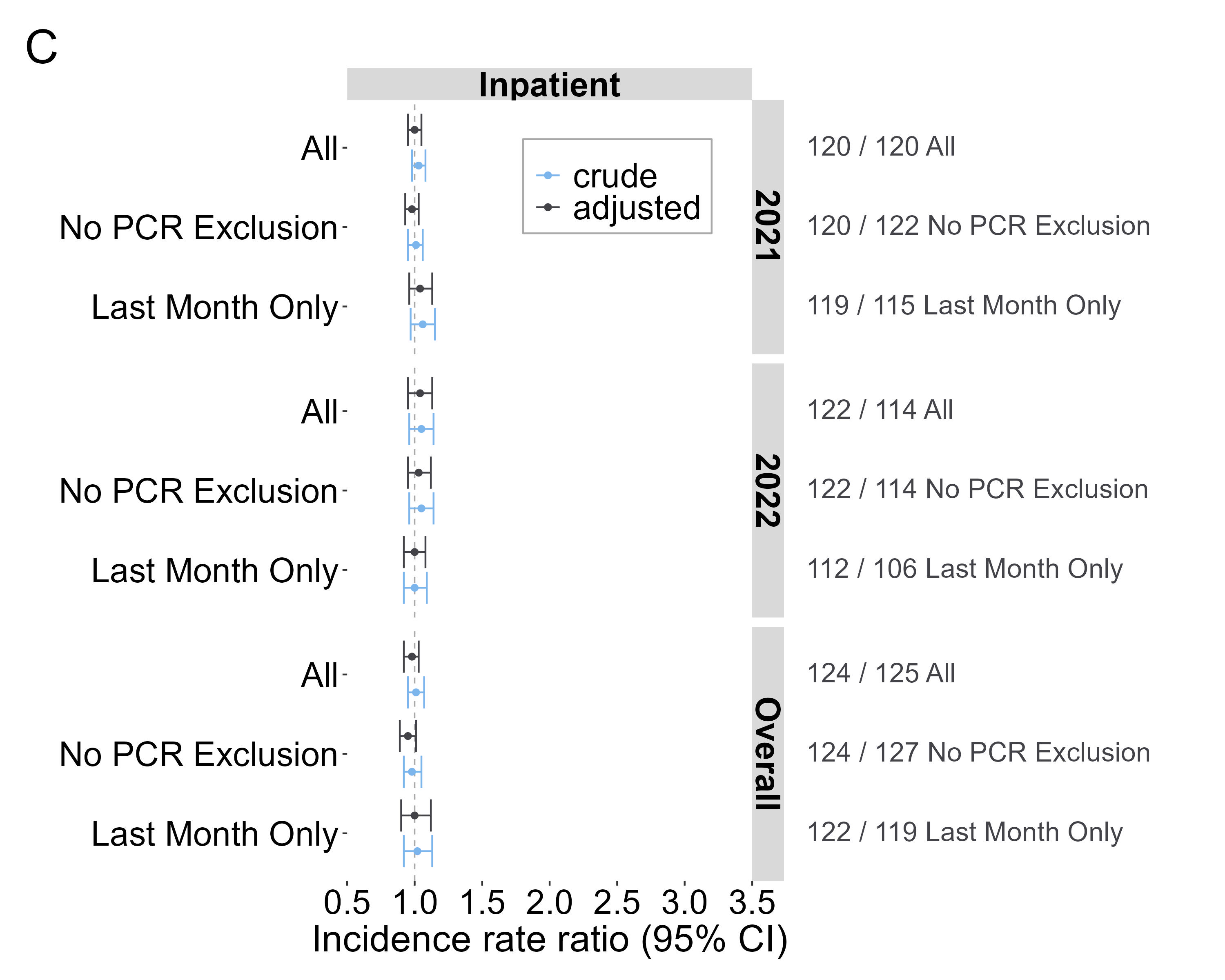

Supplement: Supplemental Material [file IANN_A_2580077_SM1394.zip › suppl_data/fig_s_2C.jpg]

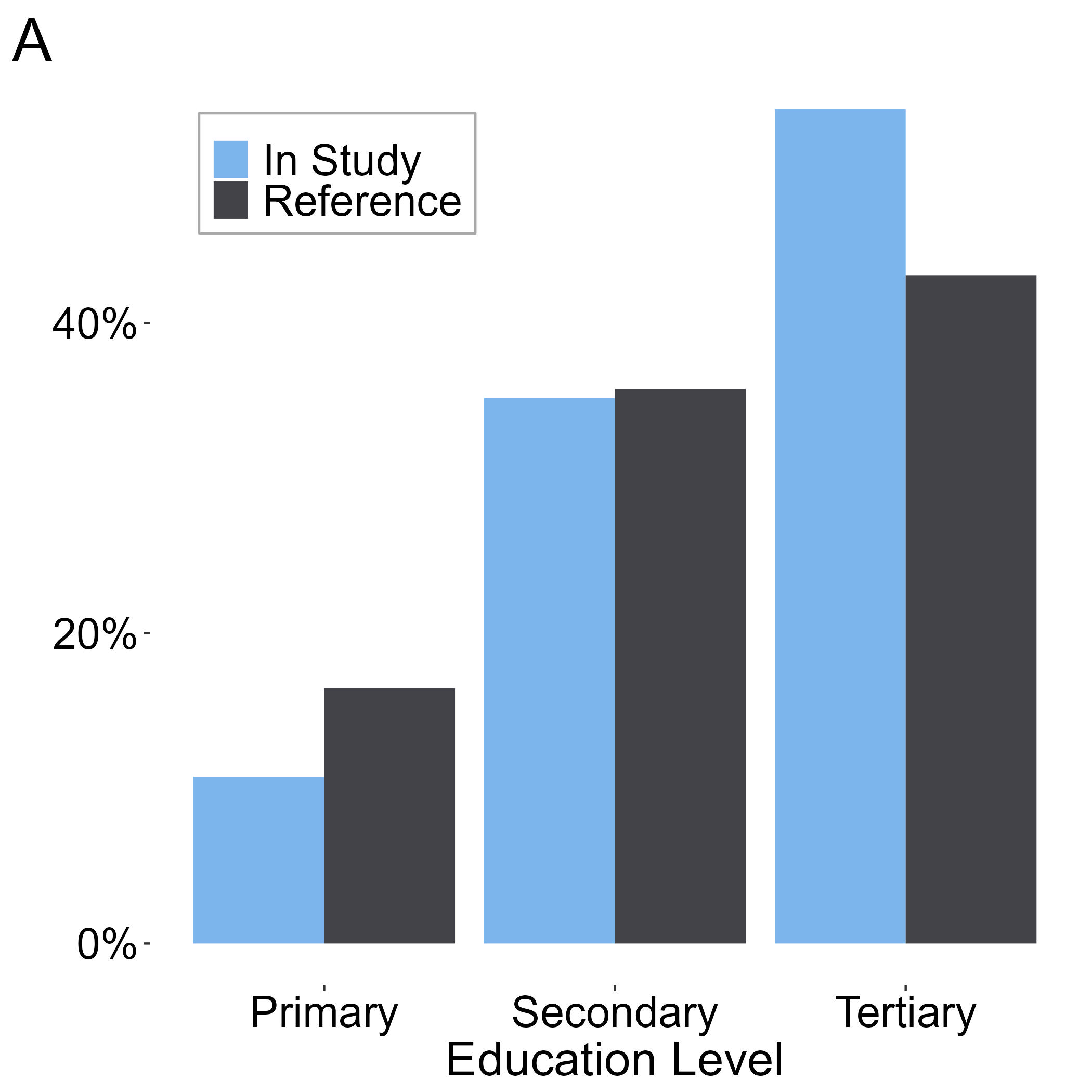

Supplement: Supplemental Material [file IANN_A_2580077_SM1394.zip › suppl_data/fig_s_3A.jpg]

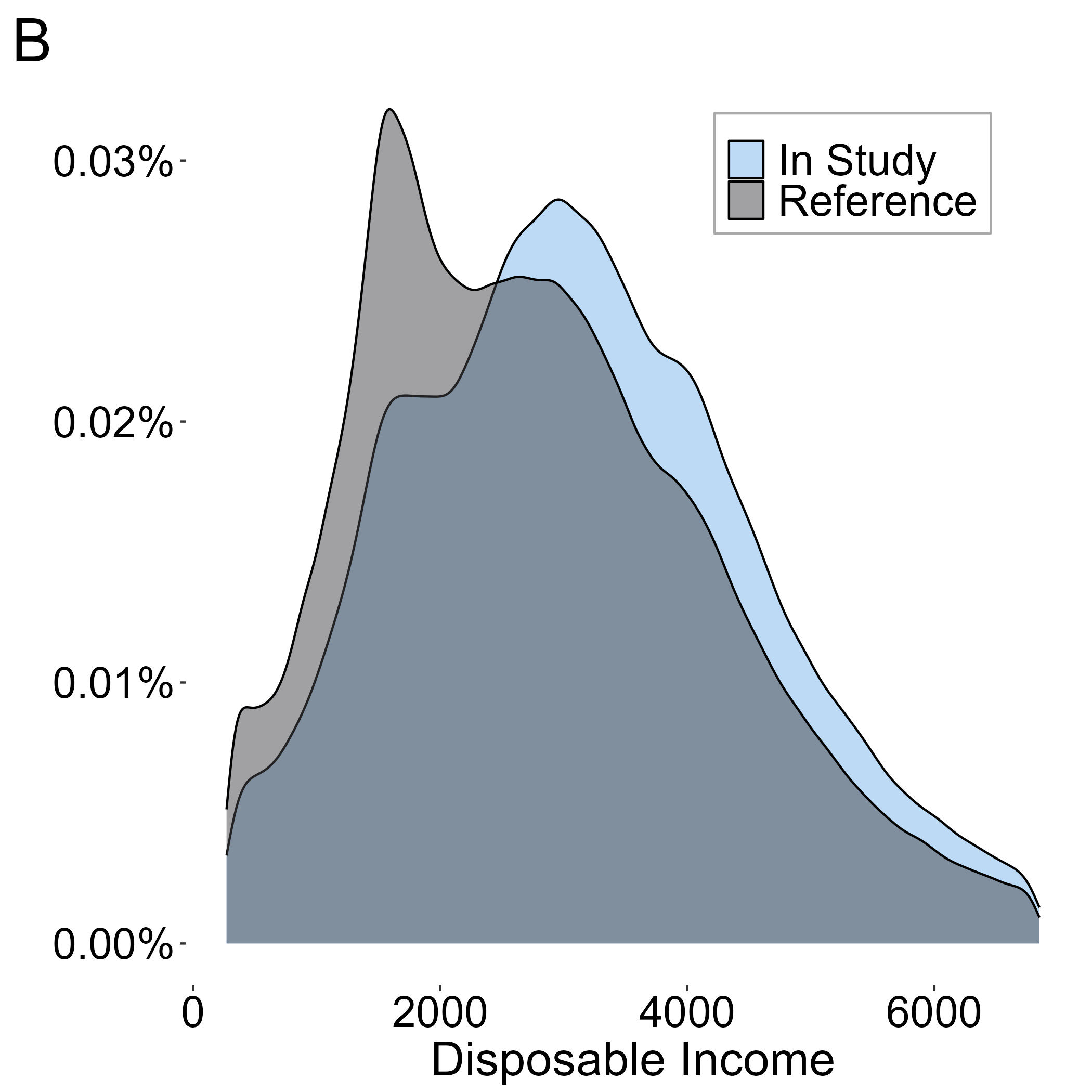

Supplement: Supplemental Material [file IANN_A_2580077_SM1394.zip › suppl_data/fig_s_3B.jpg]

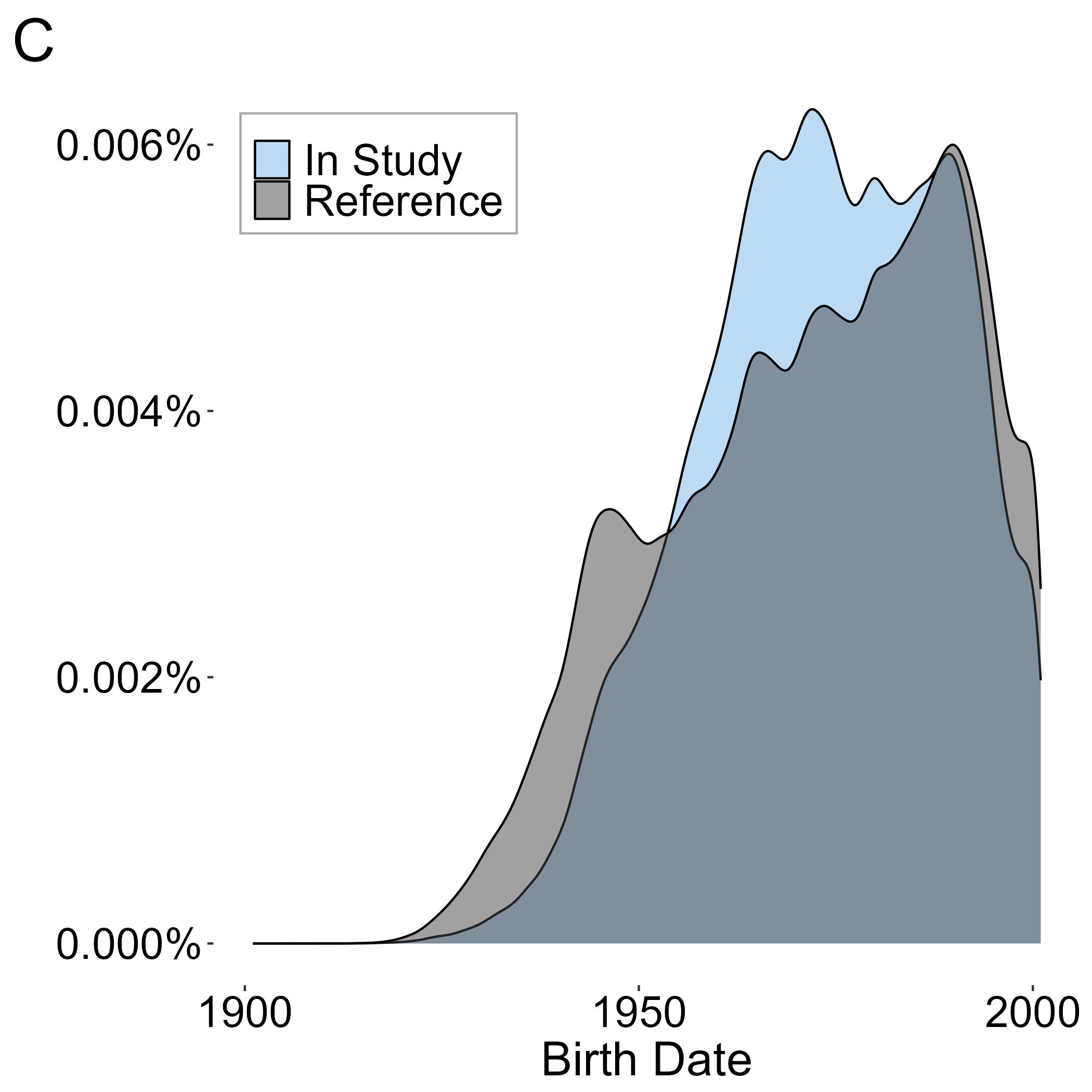

Supplement: Supplemental Material [file IANN_A_2580077_SM1394.zip › suppl_data/fig_s_3C.jpg]
